# Multitraction with a single rubber band and clips: a simple tip for endoscopic submucosal dissection of a recurrent lesion with previous clip

**DOI:** 10.1055/a-2094-9919

**Published:** 2023-06-15

**Authors:** Marion Schaefer, Jérémie Albouys, Sophie Geyl, Romain Legros, Mathieu Pioche, Jean-Baptiste Chevaux, Jérémie Jacques

**Affiliations:** 1 Department of Hepatogastroenterology, Regional University Hospital of Nancy, Nancy, France; 2 Department of Hepatogastroenterology, Dupuytren Hospital, Limoges, France; 3 Department of Endoscopy and Hepatogastroenterology, Edouard Herriot Hospital, Lyon, France; 4 BioEM, XLim, UMR 7252, CNRS, Limoges, France

Endoscopic submucosal dissection (ESD) with double-clip traction (DCT) with a rubber band and two clips allows excellent en bloc and curative resection rates and is probably the cheapest traction system available 1 . Incomplete previous resection or recurrence is associated with a higher piecemeal resection rate 2 and severe fibrosis is an independent predictor of perforation 3 . DCT is also safe and effective in these settings 4 . Multipolar traction has been reported to improve the visualization of the submucosa with four peripheral rubber bands fixed with four clips on the edges and attached to a central band that is fixed on the opposite wall with a fifth clip 5 . This is effective but requires some time to set up the device, which can be difficult to advance up through a thin operative channel.

We report the case of a 63-year-old woman with a partially resected cecal granular laterally spreading tumor (LST) and a clip placement, referred for ESD. Initial evaluation showed a 50 × 40-mm granular LST with a retractive aspect on a fold and persistence of the clip, without signs submucosal invasion ( Fig. 1 ). After submucosal injection of glycerol mixed with indigo carmine, circumferential incision, and trimming of the edges, a first clip with a rubber band attached was introduced into the scope and fixed to the medial part of the anal side of the lesion ( Video 1 ). Then two other clips were placed on either side of the first one, also gasping the elastic. Finally, the elastic was caught with a fourth clip and fixed on the opposite wall ( Fig. 2 ). This simple strategy allowed even better exposure of the submucosa, in particular in the lateral edges of the lesions, and sufficient view under the previous clip ( Fig. 3 ), which was the site of intense fibrosis, to achieve en bloc resection of the lesion without any perforation. After resection, the fold was almost erased.

**Fig. 1:**
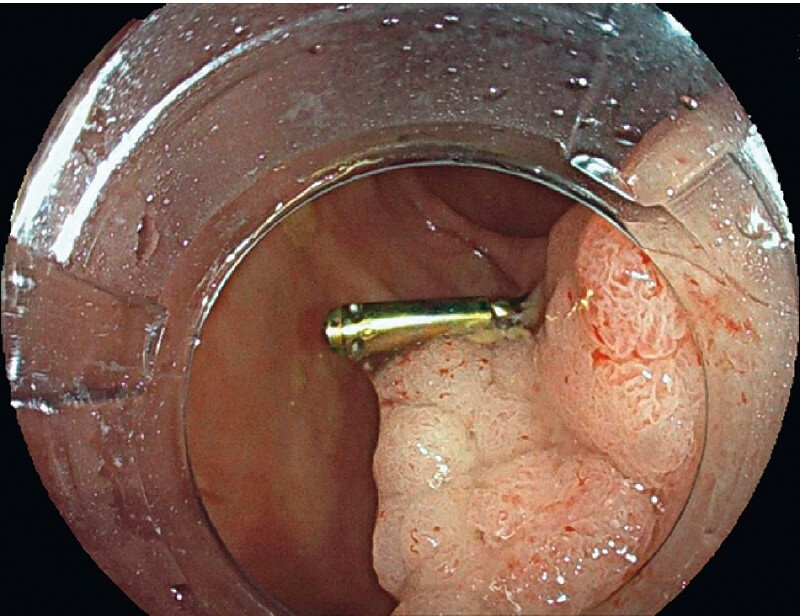
Granular laterally spreading tumor with previously placed clip.

**Video 1** Multitraction with a single rubber band and clips: a simple tip for endoscopic submucosal dissection of a recurrent lesion with previous clip.


**Fig. 2:**
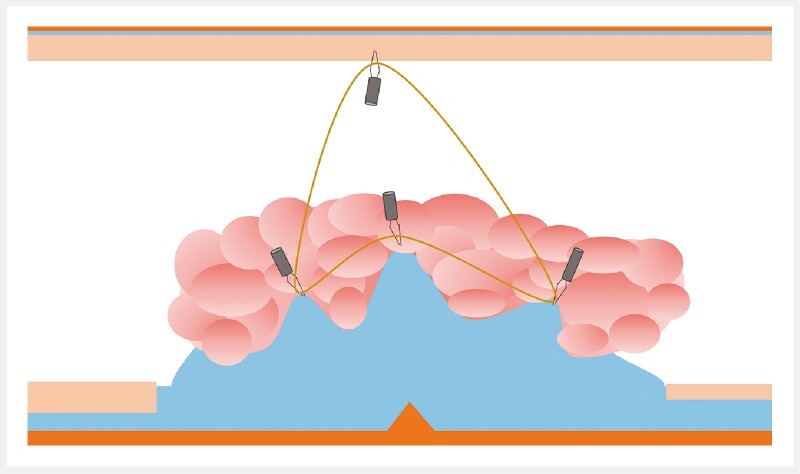
Placement of multitraction with a single rubber band.

**Fig. 3:**
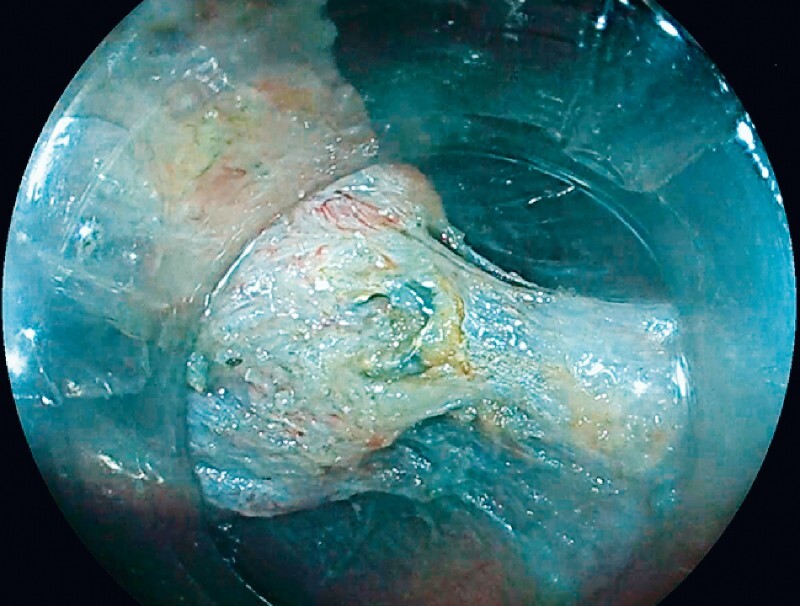
View under the previously placed clip.

Multitraction with a single rubber band is a cheap and easy-to-use technique that could help to expose lateral edges of submucosa even better in challenging cases of ESD like recurrent lesions.

Endoscopy_UCTN_Code_TTT_1AQ_2AD

## Notes

Endoscopy E-Videos

https://eref.thieme.de/e-videos
*E-Videos* is an open access online section of the journal *Endoscopy* , reporting on interesting cases and new techniques in gastroenterological endoscopy. All papers include a high-quality video and are published with a Creative Commons CC-BY license. Endoscopy E-Videos qualify for HINARI discounts and waivers and eligibility is automatically checked during the submission process. We grant 100% waivers to articles whose corresponding authors are based in Group A countries and 50% waivers to those who are based in Group B countries as classified by Research4Life (see: https://www.research4life.org/access/eligibility/ ). This section has its own submission website at https://mc.manuscriptcentral.com/e-videos
